# Modelling Parkinson’s Disease: iPSCs towards Better Understanding of Human Pathology

**DOI:** 10.3390/brainsci11030373

**Published:** 2021-03-14

**Authors:** Sahar Avazzadeh, Jara Maria Baena, Cameron Keighron, Yajaira Feller-Sanchez, Leo R. Quinlan

**Affiliations:** Physiology and CÚRAM SFI Centre for Research in Medical Devices, School of Medicine, National University of Ireland (NUI), H91 TK33 Galway, Ireland; sahar.avazzadeh@nuigalway.ie (S.A.); jaramariabaena.montes@nuigalway.ie (J.M.B.); cameron.keighron@nuigalway.ie (C.K.); yajairafeller.sanchez@nuigalway.ie (Y.F.-S.)

**Keywords:** Parkinson’s disease, induced pluripotent stem cells, human pathology

## Abstract

Parkinson’s Disease (PD) is a chronic neurodegenerative disorder characterized by motor and non-motor symptoms, among which are bradykinesia, rigidity, tremor as well as mental symptoms such as dementia. The underlying cause of Parkinson disease is degeneration of dopaminergic neurons. It has been challenging to develop an efficient animal model to accurately represent the complex phenotypes found with PD. However, it has become possible to recapitulate the myriad of phenotypes underlying the PD pathology by using human induced pluripotent stem cell (iPSC) technology. Patient-specific iPSC-derived dopaminergic neurons are available and present an opportunity to study many aspects of the PD phenotypes in a dish. In this review, we report the available data on iPSC-derived neurons derived from PD patients with identified gene mutations. Specifically, we will report on the key phenotypes of the generated iPSC-derived neurons from PD patients with different genetic background. Furthermore, we discuss the relationship these cellular phenotypes have to PD pathology and future challenges and prospects for iPSC modelling and understanding of the pathogenesis of PD.

## 1. Introduction

Parkinson’s disease (PD) is a complex, progressive neurological disorder characterized by degeneration of dopaminergic neurons (DA) in the substantia nigra pars compacta of the ventral mesencephalon [1]. The prevalence of PD is increasing with more than 6.1 million individuals reported globally to have PD in 2016 compared with 2.5 million in 1990 [2]. The increase in prevalence is due both to the ageing global population and associated changes in population behaviors such as smoking, decreased physical activity, and environmental factors such as air pollution [3,4]. PD occurrence is also sex-dependent with males displaying 1.5 times higher incidence compared to females [5]. The characteristic symptoms of PD are generally classified as motor (tremor, bradykinesia, and postural instability) and non-motor (dementia, depression, anxiety, fatigue, and pain) [6,7]. While the majority of PD cases are idiopathic without any clear family history, numerous genetic mutations have been found in individuals, with more rare and familial forms of PD also reported [8]. These genetic factors include autosomal dominant and recessive genes such as leucine-rich-repeat kinase 2 (*LRRK2*), *PARK2* (encoding Parkin), PTEN-induced putative kinase (*PINK1*), *PARK7* (encoding *DJ-1*), *SNCA* (encoding α-synuclein), and glucosidase beta acid (*GBA*). Each of these gene can be seen to present with variable clinical phenotypes.

**Figure 1:**
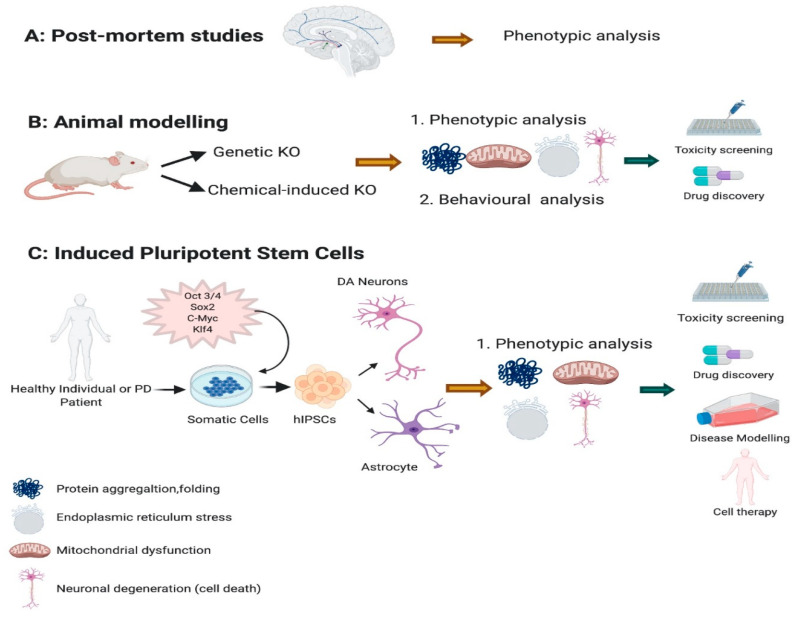
Parkinson’s disease (PD) modelling. PD have been modelled and studied using post-mortem (**A**) tissues derived from PD. (**B**) Animal models have been vastly used both by knocking down specific gene or with use of chemicals. This help researchers to investigate the associated phenotypes such as mitochondrial dysfunction, neuronal degeneration and protein folding and aggregation for creating an efficient animal model and use in drug discovery and toxicity. (**C**) Novel induced pluripotent stem cells have been derived from somatic cells of a PD or a healthy individual, leading to generate a disease model in a dish where different phenotypes can be investigated and pave the way towards drug discovery, toxicity testing and cell therapy interventions.

Previous studies have indicated that one of the main pathological features of PD is the formation of α-synuclein aggregation which leads to Lewy bodies development in both familial and sporadic PD [9,10]. Other key factors that are strongly associated with PD pathologies are oxidative stress, mitochondrial dysfunction, and neuroinflammation [11,12,13,14]. However, the precise pathogenesis of PD remains unknown. One of the major barriers in PD research is the lack of available brain tissue to study the problem in detail (Figure 1A). This has hampered investigations of the cellular and molecular mechanism underlying DA degeneration. As a substitute, DA ablation in animal models (Figure 1B) has been very useful, but as yet not fully recapitulating the complex phenotypes observed in PD [15,16]. Neurotoxins such as 6-hydroxydopamine (6-OHDA), and 1-methyl-4-phenyl-1,2,3,6-tetrahydropyridine (MPTP) have been conventionally used in PD modelling [14]. These neurotoxins have been injected into animal brains, working by inhibition of complex I of the mitochondrial electron transport chain leading to oxidative stress and eventual neuronal death [17,18]. While these toxins cause neuronal damage but it does not yield aggregation of α-synuclein which is the major pathological marker of PD. Additionally, there are significant challenges in generating genetic animal models for selective degeneration of DA neurons [19].

A viable and exciting alternative approach to modelling PD seemed inevitable with the development of human pluripotent stem cells (iPSCs) [20,21] (Figure 1C). This was particularly exciting considering iPSC-derived mesencephalic DA neurons are indistinguishable from human fetal mesencephalic DA neurons in respect to their functionality, potency, maturity, and axonal outgrowth capacity [22]. While iPSCs represent a patient’s complete genomic background and as such provide a unique platform for modelling in particular, specific genes associated with disease, the potential of iPSCs to reveal important insights on the mechanism of PD pathogenesis is limited to date.

A number of recent reviews have focused on the advantage of using iPSCs in PD modelling which have contributed extensively to our knowledge [23,24]. However, a more comprehensive and detailed study of iPSC modelling and their related phenotypes to a specific genomic background and their ultimate relation to human pathology is lacking. This review presents the latest data on the modelling of iPSC-derived DA neurons with specific mutations in the key PD associated genes *SNCA*, *LRRK2*, *PARK2*, *PINK1*, *GBA,* and *DJ-1*. We will focus on the most common associated phenotypes that are strongly correlated and connected in human PD pathology: mitochondrial dysfunction, mitochondrial DNA damage, oxidative stress, and accumulation and aggregation of α-synuclein.

## 2. iPSC Generation and Differentiation of Dopaminergic Neurons

iPSC technology involves the conversion of human somatic cells into proliferative pluripotent stem cells, essentially equivalent to human embryonic stem cells [21]. The initial discovery was reported 15 years ago using mouse embryonic fibroblasts using the forced expression of so-called Yamanaka factors (Oct4, Sox2, Klf4, c-myc) [25]. The following year the same group further advanced this approach when they demonstrated that adult human dermal fibroblasts could also be reprogrammed into iPSCs [26]. These studies revolutionized stem cell research, making it possible to consider the use of patient-specific iPSC-derived cells to treat and/or investigate a myriad of human diseases.

In the context of PD, considering the loss of DA neurons as the primary pathological hallmark, the initial goal with iPSCs was to develop differentiation protocols to generate a homogenous population of tyrosine hydroxylase-positive (TH^+^) dopaminergic neurons. This has been achieved using numerous different approaches (reviewed in [27,28]), such as forced expression of the transcription factors ASCL1, NURR1 and LMX1A [29], dual SMAD inhibition-based floor plate (FP) protocols [30,31,32] or transient expression of transcription factors by means of AVV vectors [32]. To date more than 385 iPSC-derived neuronal lines from PD patients with different genetic mutations have been successfully generated across many independent laboratories all over the world (reviewed in [33]). In this review we synthesize the available data on phenotype characterization as the starting point in securing the iPSC platform for expanding a new paradigm for cellular therapy approaches and drug development and screening for both preclinical and clinical studies.

## 3. Modelling PD Using iPSCs

There has been remarkable advances in the discovery of genetic mutations associated with PD in recent years [34]. Familial PD collectively accounts for only 10% of all PD cases while the remainder of PD cases have unknown etiology [35,36]. Patient-specific iPSC-derived DA neurons with specific mutations allows the underlying mechanisms of a particular mutation to be investigated in depth. Here, we report on the available data based only on human iPSC-derived neuronal models, investigating specific mutation-associated phenotypes of PD.

### 3.1. iPSC Modelling of SNCA Mutation and Associated Phenotypes

The first reported mutation associated with autosomal-dominant PD was that of α-synuclein [37]. α-synuclein is involved in numerous important cellular functions such as modulation of intracellular vesicle trafficking [38], dopamine metabolism [39], microtubule nucleation and proliferation [40], as well as the regulation of synaptic vesicle recycling [41]. There are numerous specific mutations associated with *SNCA*-related PD pathology including A30P, G51D, E46K, A53T, and A53E [42,43,44,45]. The severity of symptoms for PD is proportional to the number of *SNCA* copies deleted. In human iPSC-derived neurons the most commonly studied alterations in *SNCA* are the A53T mutation and multiplication of *SNCA* as they are the most common mutations associated with PD. Alterations in α-synuclein physiology results in a myriad of cellular changes must commonly mediated through mitochondrial dysfunction and oxidative stress. These changes and abnormal aggregation of α-synuclein not only induce neuronal loss but are also seen to prevent neuronal regeneration.

#### 3.1.1. SNCA Alteration Disrupts Normal Mitochondrial Function in iPSC-Derived Neurons

**Figure 2:**
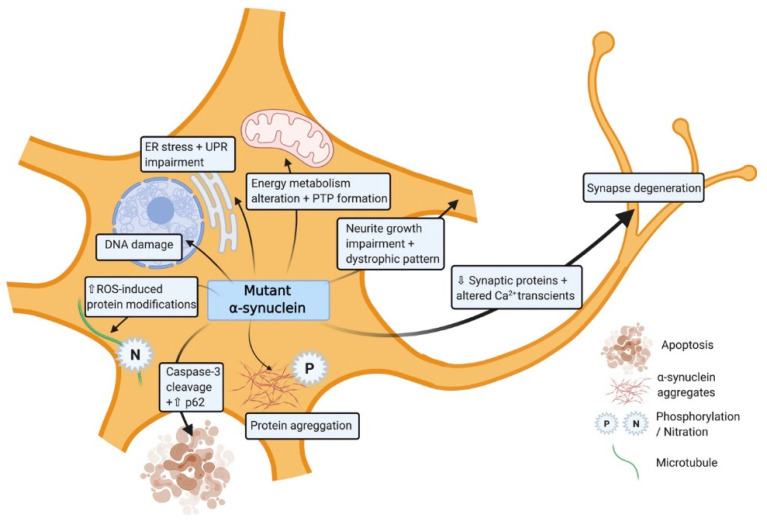
Induced pluripotent stem cell (iPSC)-derived dopaminergic neurons modelling the role of mutant *SNCA*. The illustration shows the impact of the mutated α-synuclein in different cellular processes within the cell.

Mitochondrial impairment is very common in all *SNCA* altered iPSC-derived neurons [46,47] (Table 1). For *SNCA*-triplication iPSC-derived neuronal progenitors (NPCs), mitochondrial dysfunction presents as altered energy metabolism associated with impaired basal and/or maximal respiration capacity, or ATP production in [48] (Figure 2). This is also seen in *SNCA*-A53T iPSC-derived neuroepithelial stem cells and neurons [46,49,50]. Certain pathologies result in structures such as permeability transition pores (PTP) appearing in the inner membrane of damaged mitochondria. In *SNCA*-triplication iPSC-derived neurons exposed to low concentrations of ferutinin or laser-induced ROS, suggesting *SNCA* alterations results in higher susceptibility to PTP formation in comparison to controls [51]. In addition, mitochondrial axonal transport is decreased in *SNCA*-duplication and also in A53T iPSC-derived neurons leading to energy deficits and synapse degeneration [52,53]. Upregulation of Miro1, a key protein in mitochondrial transport has also been detected in *SNCA*-A53T iPSC-derived neurons, suggesting mitophagy delay [54]. Furthermore, mitochondrial morphology is also altered in mutated neurons, manifesting as more circular and unbranched in structure with significant reductions in mitochondrial membrane potential [50].

Several studies have demonstrated that *SNCA* mutated neurons have increased sensitivity to mitochondrial toxin-induced oxidative stress [49,54] which can be further aggravated by metal ion interactions [55]. Furthermore, the expression level of oxidative stress markers such as DNAJA1, HMOX2, UCHL1, and HSPB1 which have neuroprotective capacity are also significantly dysregulated [56]. In line with this endogenous antioxidant pathways are elevated through increase activity of catalase or PGC-1α as a compensatory mechanism in response to the oxidative stress [50]. *SNCA*-A53T iPSC-derived neurons also generate higher levels of nitrous oxide (NO) after exposure to low levels of agrichemicals, resulting in disruption of microtubules [52] (Figure 2). These data support the growing evidence that exposure to certain environmental agents can significantly contribute to the PD pathology in those with alterations in *SNCA*.

**Table 1:** *SNCA*-mutated iPSC-derived neuronal phenotypes.

Reference	Number of Cohorts	Type of Mutation	Cell Type	Phenotype
[48]	1 PD line vs. 2 control lines	Autosomal dominant Triplication	iPSC-derived neuronal progenitor cells	1. Elevated α-synuclein levels 2. Decreased neuronal activity 3. Increased autophagy 4. Mitochondrial dysfunction 5. Increased oxidative stress
[57]	1 PD line vs. 1 control line	Autosomal dominant Triplication	iPSC-derived cortical neurons	1. Elevated α-synuclein levels 2. Endoplasmic Reticulum stress
[58]	1 PD line vs. 2 control lines	Autosomal dominant Triplication	iPSC-derived DA neurons	1. Elevated α-synuclein levels 2. Impairment in neuronal development 3. Impairment in synaptic transmission 3. Increased autophagy
[47]	1 PD line vs. 1 control line	Autosomal dominant Duplication	iPSC-derived midbrain DA and Cortical projection neurons	1. Elevated α-synuclein levels 2. Increased α-synuclein aggregation 3. Increased phosphorylated α-synuclein 4. Increased oxidative stress
[56]	1 PD line vs. 1 control line	Autosomal dominant Triplication	iPSC-derived DA neurons	1. Increased α-synuclein aggregation 2. Increased oxidative stress
[55]	1 PD line vs. 1 control line	Autosomal dominant Triplication	iPSC-derived cortical neurons	1. Elevated α-synuclein 2. Increased oxidative stress
[59]	1 PD line	Autosomal dominant Triplication	iPSC-derived DA progenitor cells	1. Elevated α-synuclein levels 2. Increased oxidative stress 3. Increased cell death
[60]	1 PD line vs. 1 control line	Autosomal dominant Triplication	iPSC-derived DA neurons	1. Elevated α-synuclein levels 2. Altered Calcium signalling
[61]	1 PD line vs. 1 control line	Autosomal dominant Triplication	iPSC-derived DA and basal forebrain cholinergic neurons	1. Elevated α-synuclein levels 2. Increased α-synuclein aggregation 3. Increased DNA damage
[62]	1 PD line vs. 3 control lines	Autosomal dominant Triplication	iPSC-derived DA neurons	1. Elevated α-synuclein levels 2. Impairment in neuronal development 3. Increased α-synuclein phosphorylation 4. Increased cell death and apoptosis
[63]	1 PD line vs. 1 control line	Autosomal dominant Triplication	iPSC-derived neuronal progenitor cells	1. Elevated α-synuclein levels 2. Increased DNA damage
[51]	1 PD line vs. 1 control line vs. 1 isogenic control line	Autosomal dominant Triplication	iPSC-derived cortical neurons	1. Elevated α-synuclein levels 2. Mitochondrial dysfunction
[53]	1 PD line vs. 2 control line	Autosomal dominant Duplication	iPSC-derived cortical forebrain glutamatergic neurons	1. Elevated α-synuclein levels 2. Increased α-synuclein aggregation 3. Mitochondrial transport impairment
[49]	1 PD line vs. 1 isogenic control line	Autosomal dominant A53T	iPSC-derived A9 DA neurons	1. Mitochondrial dysfunction 2. Increased oxidative stress 3. Increased cell death and apoptosis 4. Neuronal maturation impairment
[46]	2 PD lines vs. 1 control line	Autosomal dominant A53T and A30T	iPSC-derived neural stem cells	1. Mitochondrial dysfunction
[64]	2 PD line vs. 1 control line	Autosomal dominant A53T	iPSC-derived DA, GABAergic and glutaminergic neurons	1. Altered synaptic activity 2. Increase α-synuclein aggregation 3. Impairment in Neuronal development
[52]	1 PD line vs. 1 isogenic control line	Autosomal dominant A53T	iPSC-derived A9 DA neurons	1. Mitochondrial transport impairment 2. Alteration in microtubules function
[50]	4 PD lines vs. 3 control line	Autosomal dominant A53T and Triplication	iPSC-derived DA neurons	1. Elevated α-synuclein levels 2. Endoplasmic Reticulum stress 3. Mitochondrial dysfunction 5. Increased autophagy 6. Increased oxidative stress
[65]	1 PD line vs. 1 isogenic control line	Autosomal dominant A53T	iPSC-derived DA neurons	1. Increased α-synuclein aggregation
[66]	1 PD line vs. 1 control line	Autosomal dominant A53T	iPSC-derived DA neurons	1. Elevated α-synuclein levels 2. Increased α-synuclein aggregation 3. Impairment in Neuronal development
[54]	3 PD lines vs. 3 control lines	Autosomal dominant A53T and triplication	iPSC-derived DA neurons	1. Elevated α-synuclein levels 2. Mitophagy impairment 3. Increased oxidative stress
[67]	2 PD lines vs. 1 control line vs. 1 isogenic control line	Autosomal dominant A53T and triplication	iPSC-derived DA neurons	1. Lysosomal dysfunction 2. Increased α-synuclein aggregation

#### 3.1.2. SNCA Alteration Leads to Protein Aggregation and Cellular Damage in iPSC-Derived Neurons

Lewy bodies formation as a consequence of the abnormal aggregation of α-synuclein is a major hallmark in human PD pathology and more specifically to DA neurons [62]. iPSC-derived neurons with *SNCA* mutations also exhibit higher levels of α-synuclein phosphorylation and increased α-synuclein aggregate formation (Figure 2; Table 2). In A53T iPSC-derived neurons reports describe posttranslational change in α-synuclein, such as Ser129 phosphorylation or ubiquitination leading to the formation of large aggregates and Lewy Bodies [64,66]. In addition, A53T mutations result in increased interactions of elevated α-synuclein levels with essential transcription factors, ribonucleoproteins, and ribosomal proteins [49,66]. This aggregation instigates a dysregulation in protein production and transcription-related mRNAs in *SNCA*-A53T iPSC-derived neurons [60,65,68]. This excessive accumulation of aberrant misfolded protein aggregates within the cell results in endoplasmic reticulum stress and activation of the unfolded protein response (UPR) which is also observed in both *SNCA*-triplication and A53T iPSC-derived neurons (Figure 2) [50,57]. A reduction in the level of a key factor in UPR pathway IREα, supports the damaging consequences of these aggregations in *SNCA*-triplication and A53T iPSC-derived neurons [50,57]. In the related lysosomal stress pathway, α-synuclein deactivation of the SNARE protein ykt6 leads to an impaired physiological response to lysosomal stress, in *SNCA* mutated iPSC-derived neurons [67]. The exposure of *SNCA*-triplication iPSC-derived neurons to toxins results in an elevation in cell death, caspase-3 cleavage [62] and the presence of autophagosomes [58]. This suggest that iPSC-derived neurons with *SNCA* mutations show higher vulnerability to toxins and undergo apoptosis. Intron 1 methylation of *SNCA*-triplication gene has shown to rescue this phenotype and improve cell viability [59]. Similar processes have been reported in *SNCA*-A53T iPSC-derived neurons by an increase in autophagy-related proteins such as p62 or the autophagosome marked LC3 after exposure to agrochemicals [49,50]. α-synuclein aggregation not only induces neuronal loss but also prevents neuronal regeneration. Significant downregulation in specific DA differentiation genes such as *DLK*, *GABABR2*, *NURR1,* and *TH* have been reported in *SNCA*-A53T iPSC-derived neurons [58]. Furthermore neurite growth, length are impaired with appearance of dystrophic neurite patterns in *SNCA*-triplication iPSC-derived neurons [66]. Neurite formation at the early stages present with many degeneration features such as swollen varicosities and spheroid inclusions leading to reduction and alteration of the number of synaptic contacts and activity respectively in *SNCA*-A53T iPSC-derived neurons [64]. Spontaneous Ca^2+^ transients with larger mean amplitude have been shown as a consequence of impairment in synaptic activity [66].

### 3.2. iPSC Modelling of LRKK2 and Associated Phenotypes

*LRRK2* is a multi-domain protein exhibiting both kinase and GTPase functions located in the *PARK8* locus on chromosome 12 [69]. *LRRK2* is implicated as a significant genetic contributor to the development of autosomal dominant familial PD as well as some cases of idiopathic PD [70,71]. To date around 20 different *LRRK2* mutations have been linked to PD pathophysiology [72]. *LRRK2* is susceptible to several missense mutations including the *LRRK2* G2019S, I2020T, Y1699C, and R1441C heterozygous mutations [73,74]. G2019S is the most commonly occurring mutation and is associated with 4% of familial PD and 1% of idiopathic PD cases [75]. *LRKK2* is recognized to have pleiotropic roles across multiple domains including neurite outgrowth [76], modulation of synaptic vesicle endocytosis [77,78] and mitochondrial function and mitophagy [79,80].

#### 3.2.1. LRKK2 Alterations Result in Mitochondrial Dysfunction in iPSC-Derived Neurons

Defective mitochondria are found to accumulate in the axons of *LRRK2* mutated iPSC-derived DA neurons as a result of disruption in mitophagy [81]. In addition, there is increased level of mitochondrial DNA in *LRRK2* R1441C iPSC-derived neurons compared to control neurons [82]. Mitochondrial impairments have also been observed in *LRRK2* G2019S human neuroepithelial stem cells (NESCs), suggesting a defective mechanism at earlier stages of neuronal development [83]. Furthermore, *LRRK2*-mutated iPSC-derived neurons show higher mitochondria mobility including more bidirectional movement, suggesting that *LRRK2* mutations can actively encourage mitochondrial evasion of mitophagy [84]. One study reports that defects in mitochondrial biogenesis and energetics are associated with low levels of nicotinamide adenine dinucleotide (NAD^+^) in *LRRK2*-mutated iPSC-derived neurons [85].

#### 3.2.2. LRKK2 Alteration Promotes α-Synuclein Aggregation in iPSC-Derived Neurons

Data from G2019S-mutated DA neurons have indicated to a role for LRKK2 in α-synuclein pathology, resulting in increased endogenous α-synuclein aggregation [86,87], accelerating neuronal loss [88]. Accumulation of α-synuclein additionally affects *LRRK2* gain of function mutations [79] suggesting that *LRRK2* mutations may confer increased susceptibility to PD through *SNCA* [89]. In addition, iPSC-derived astrocytes with *LRRK2* mutations display increased α-synuclein aggregation, leading to cell death [90]. Chemical amelioration of chaperone mediated autophagy has been shown to rescue astrocytes and DA neurons via the clearance of α-synuclein [90].

iPSC-derived DA neurons with *LRRK2* G2019S and R1441C mutations have impaired development and differentiation capability [91,92,93,94,95,96,97,98]. *LRRK2* mutated DA neurons have additionally altered neurite outgrowth and aggregation of microtubules as well as altered calcium dynamics in vitro [99]. Studies have continually implicated endo-lysosomal system dysfunction in PD pathogenesis with the serine/threonine kinase activity of lark2 as a key factor in endocytosis of synaptic vesicles [100]. iPSC ventral midbrain neurons with G2019S mutations have impaired endocytosis [101]. Consistent with this, central endocytosis proteins including dynamin-1, and various Rab proteins are significantly downregulated in iPSC-derived neurons [101,102]. Downregulation of these proteins is central to the endocytosis pathways, leading to defective clathrin-mediated synaptic vesicle endocytosis that may confer PD pathogenesis by dysregulation in these pathways [102].

**Table 2:** LRRK2-mutated iPSC-derived neuronal phenotypes.

Reference	Number of Cohorts	Type of Mutation	Cell Type	Phenotype
[88]	1 PD and isogenic KO line vs. isogenic controls	LG2019S	iPSC-derived cortical neurons	1. Increased neuronal degeneration 2. Degeneration-associated neuroinflammation
[83]	3 PD and 2 isogenic KO lines vs. 4 controls and isogenic lines	G2019S	iPSC-derived neural stem cells	1. Deficient dopaminergic differentiation 2. Mitochondrial dysfunction 3. Increased cell death
[91]	3 PD lines vs. 3 control lines	G2019S	iPSC-derived DA neurons	1. Impairment in neuronal development
[102]	8 PD lines vs. 4 control lines	G2019S and R1441C	iPSC-derived DA neurons	1. Decreased Endocytosis
[84]	2 PD lines vs. 2 control lines	G2019S, R1441C	iPSC-derived DA neurons	1. Mitochondrial dysfunction.
[62]	6 PD lines vs. 3 control lines	G2019S	iPSC-derived DA neurons	1. Increased oxidative stress 2. Increased neuronal degeneration 3. Increased α-synuclein aggregation
[90]	2 PD lines vs. 3 control lines vs. 1 isogenic control line	G2019S,	iPSC-derived DA neurons	1. Increased neuronal degeneration
[96]	4 PD lines vs. 4 control lines	G2019S	iPSC-derived DA neurons	1. Increased neuronal degeneration 2. Increased α-synuclein aggregation 3. Increased autophagy
[81]	6 PD lines vs. 3 control lines	G2019S	iPSC-derived DA neurons	1. Decreased neuronal development 2. Increased neuronal degeneration
[88]	3 PD lines vs. 3 control lines	G2019S	iPSC-derived DA organoids	1. Mitophagy impairment
[103]	1 PD line	G2019S	iPSC-derived neural stem cells	1. Increased α-synuclein aggregation
[92]	8 PD lines vs. 5 control lines vs. 4 gene edited controls	G2019S	iPSC-derived DA neurons	1. Increased mitochondrial dysfunction 2. Increased oxidative Stress
[97]	2 PD lines vs. 2 control and H1, H9 lines	G2019S	iPSC-derived neural stem cells	1. Altered calcium signalling 2. Impaired neuronal development
[93]	4 PD lines vs. 4 control line	G2019S	iPSC-derived DA neurons	1. Nuclear envelope impairment 2. Increased proteasome stress
[94]	3 PD lines vs. 1 control line	G2019S	iPSC-derived DA neurons	1. Impaired neuronal development 2. Decreased mitophagy
[87]	3 PD lines vs. 4 control lines	G2019S	iPSC-derived DA neurons	1. Impaired neuronal Development
[104]	4 PD lines vs. 7 control lines	G2019S	iPSC-derived DA neurons	1. Increased α-synuclein aggregation 2. Increased oxidative stress
[95]	1 PD and 1 isogenic KO line vs. 1 control and 1 isogenic control line	G2019S	iPSC-derived DA neurons	1. Increased apoptosis and neuronal cell death 2. Decreased mitosis
[80]	2 PD lines vs. 4 control lines	G2019S	iPSC-derived DA neurons	1. Impaired Neuronal development 2. Decreased α-synuclein phosphorylation
[82]	12 PD lines vs. 3 control lines	G2019S, R1441C	iPSC-derived DA neurons	1. Increased Tau and α-synuclein aggregation. 2. Impaired Neuronal development
[99]	3 PD lines vs. 3 control lines	G2019S	iPSC-derived sensory and DA neurons	1. Mitochondrial dysfunction
[85]	3 PD lines vs. 3 control lines	G2019S	iPSC-derived sensory, Glutamatergic and DA neurons	1. Large microtubule-containing neurite aggregations 2. Altered calcium signalling
[83]	3 PD and 2 isogenic KO lines vs. 4 control lines and 2 isogenic control	G2019S	iPSC-derived neural stem cells	1. Mitochondrial Dysfunction
[105]	2 PD lines vs. 2 control lines	G2019S	iPSC-derived DA neurons	1. Mitochondrial Dysfunction 2. Altered mitophagy

### 3.3. iPSC Modelling of PARK2 Mutations and Associated Phenotypes

Parkin is an E3 ubiquitin ligase, residing in the cytosol, that functions in the ubiquitin proteasome pathway [106,107]. *PARK2* which is located on the 6q25.2–27 chromosome encodes parkin and is the most frequent gene mutation associated with autosomal recessive early onset familial PD [107]. Fifty percent of all PD cases under the age of 45 are associated with parkin mutations [108] (Table 3). Mutations in parkin range from small deletions and base pair substitution to large deletions spanning hundreds of nucleotides [109]. Parkin has been primarily shown to be important in maintaining normal mitochondrial function and integrity [110].

#### PARKIN Mutations Result in Mitochondrial Dysfunction and Oxidative Stress in iPSC-Derived Neurons

Mitochondrial dysfunction, abnormal morphology, and impaired mitochondrial homeostasis are some of the key features displayed by parkin iPSC-derived DA neurons. These neurons exhibit swollen cristae and a highly condensed matrix in the inner mitochondrial membrane (IMM), with abnormal mitochondrial morphology directly affecting function [111] as well as an elevation in the number of enlarged mitochondria [112,113]. Pyruvate kinase M and 14-3-3 epsilon are among the most dysregulated mitochondrial proteins associated with parkin mutated neurons, and this pair have also been consistently shown to be changed in post mortem brain tissues of PD patients [113,114,115].

**Figure 3:**
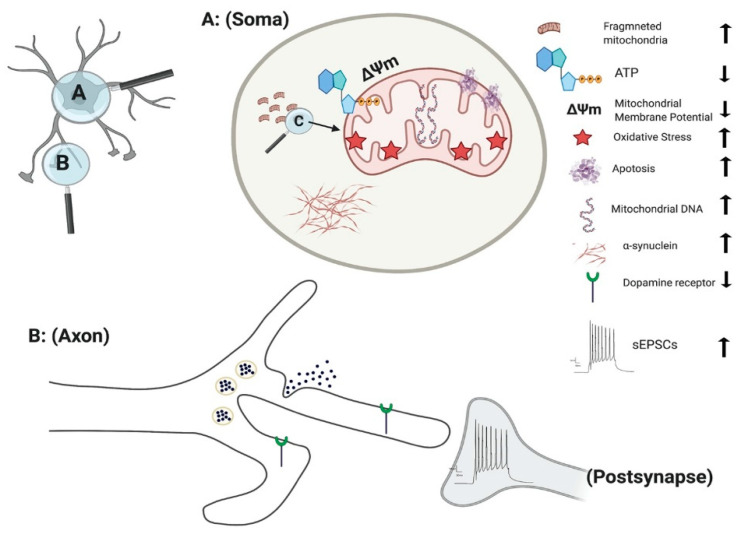
Effect of *PINK1* and parkin mutation in IPSC-derived neurons. Parkin mutation in iPSC-derived neurons showed impairment in their requitement to *PINK1*, alteration in spontaneous postsynaptic current activity, reduction in dopamine receptor and release. *PINK1* mutation in iPSC-derived neurons displayed fragmented mitochondria with alteration their DNA level, ATP, membrane potential and oxidative stress. Additionally, there is an increase in apoptotic cell death and α-synuclein aggregation and accumulation.

The culmination of mitochondrial dysfunction is thought to be an increase in oxidative stress, leading to dopamine oxidation [116]. Under normal physiological conditions the transcription of mitochondrial enzymes such as monoamine oxidases (MAO) A and B are limited, as parkin suppresses dopamine-induced oxidative stress [117,118]. However, the levels of MAO-A and B were found to be significantly increased in parkin mutated iPSC-derived DA neurons, suggesting an escalation in dopamine-induced oxidative stress [118]. Increased oxidative stress is a consistent factor observed in numerous independent studies of parkin mutations [111,112,119]. Furthermore, anti-oxidative proteins are significantly reduced in parkin mutated iPSC-derived DA neurons [120]. Conversely, Nrf2, a protein promoting antioxidant gene expression is significantly enhanced in parkin mutated iPSC-derived neurons [111]. Related to this oxidative stress inducing environment, there is a marked decrease in dopamine uptake and increased levels of spontaneous dopamine release in iPSC-derived DA neurons with parkin mutations [121]. This increased spontaneous leak of dopamine is observed in both heterozygous and homozygous forms of parkin neurons, independent of intracellular Ca^2+^ [121] (Figure 3). Additionally, the number of correctly folded and trafficked dopamine transporters (DAT) are significantly decreased [121]. Thus, parkin is presumed to mitigate dopamine oxidation and control the transmission of dopamine. A further study has demonstrated that the activation of dopamine D1-class receptors in parkin neurons leads to large rhythmic outbursts of spontaneous excitatory postsynaptic currents (sEPSCs) [122] (Figure 3). These rhythmic outburst of sEPSCs resemble oscillatory activities observed within basal ganglia neurons in PD. Overexpression of parkin in this same study resulted in a significant rescue of iPSC-derived neurons, returning oscillatory activities to normal levels [122]. These data show that parkin mutations enhance abnormal dopaminergic modulation and release in neurons.

**Table 3:** PARK2-mutated iPSC-derived neuronal phenotypes.

Reference	Number of Cohorts	Type of Mutation	Cell Type	Phenotype
[119]	6 PD patient lines vs. 3 control lines	Exon 2–4 or 6–7 deletions	iPSC-derived DA neurons	1. Increased Oxidative stress 2. Mitophagy impairment
[122]	3 PD patient lines vs. 3 control lines	Exon 3–5 or R42P deletions	iPSC-derived DA neurons	1. Dopamine dysregulation
[111]	2 PD patient lines vs. 2 control lines	Exon 2–4 or Exon 6–7 deletions	iPSC-derived DA neurons	1. Increased oxidative stress 2. Mitochondrial dysfunction 3. Increase α-synuclein aggregation
[123]	4 PD patient lines with 1 control line	Exon 3–4, R275W or R42P deletions	iPSC-derived DA neurons	1. Mitochondrial dysfunction 2. Increase α-synuclein aggregation
[124]	1 PD patient line vs. 1 control line	Del202-203AG and IVS1+1G/A	iPSC-derived DA neurons	1. Increased cell death
[113]	2 Isogenic mutated PD lines vs. 1 control line	Exon 2 deletion	iPSC-derived DA neurons	1. Mitochondrial dysfunction
[118]	2 PD lines vs. 2 control lines	Exon 4 deletion	iPSC-derived DA neurons	1. Dopamine dysregulation 2. Increased oxidative stress
[125]	2 PD lines vs. 2 control lines	Exon 2–4 deletion	iPSC-derived DA neurons	1. Mitochondrial dysfunction
[126]	2 Isogenic mutated iPSC lines vs. 1 control line	Exon 2 deletion	iPSC-derived DA neurons	1. Lysosomal dysfunction
[120]	1 PD line vs. 1 control line	Exon 5 deletion	iPSC-derived DA neurons	1. Increase α-synuclein aggregation 2. Reduced level of anti-oxidative proteins
[127]	3 PD lines vs. 3 control lines	Exon 7 deletion, c.1072delT or Exon 1 deletion and c.924C>Tor c.1072delT	iPSC-derived DA neurons	1. Mitochondrial dysfunction
[112]	2 PD lines vs. 2 control lines	c.1366C.T and c.1072Tdel	iPSC-derived DA neurons	1. Dopamine dysregulation 2. Increase cell death 3. Increase α-synuclein aggregation

### 3.4. iPSC Modelling of PINK1 Mutations and Associated Phenotypes

Phosphatase and tensin homolog (PTEN)-induced putative kinase 1 (*PINK1*) mutations are the second most frequent cause of early-onset PD and is involved in an autosomal recessive familial form of PD [128]. *PINK1*-related PD usually appears in the third or fourth decade of life, and like other recessive early onset forms, presents as a slow progression of the disease with a consistent response to levodopa treatment [129]. Forty-two different mutations have been found within the exons of the *PINK*1 in both heterozygous and homozygous states with Q456X is the most prevalent form of *PINK1*-related PD mutation [130,131] (Table 4). *PINK1* consists of a C-terminal kinase domain and a mitochondrial targeting sequence at the N-terminus. Cytosolic *PINK1* is released by truncation of the N-terminal portion of the gene in a proteasome-dependent manner [132,133]. *PINK1* has been shown to have an essential role in mitochondrial function, calcium homeostasis, autophagy/mitophagy, protection from stress, and protein misfold [134,135,136].

#### PINK1 Mutations Result in Loss of Mitochondrial Function and Increases Reactive Oxygen Species Generation in iPSC-Derived Neurons

*PINK1* mutations are thought to impair mitochondrial function due to a loss of function, based on the upregulation of PGC-1α and an increase in mitochondrial DNA (mtDNA) copy number (Figure 3) [137]. Under normal physiological conditions, mitochondrial damage activates *PINK1* kinase activity, and activated *PINK1* phosphorylates ubiquitin at a conserved residue of Ser65 [138]. Parkin cooperates with *PINK1* in the phosphorylation process, preparing the damaged mitochondria for lysosomal and proteasomal targeted degradation [139]. Pathogenic mtDNA mutations are found widely in individuals with PD, resulting in mitochondrial dysfunction. Siebler et al., demonstrated that while the level of mtDNA is decreased in wild-type neurons, it remained unchanged in *PINK1^−/−^* iPSC-derived neurons upon mitochondrial depolarization, suggesting an increase accumulation of mitochondria DNA dues to loss of PINK function [137]. *PINK1^+/−^* iPSC-derived neurons show a significant number of cells with fragmented mitochondria suggestion an alteration in mitochondrial cycling dynamics towards increased organelle fission [140] (Figure 3).

In addition, iPSC-derived neurons with *PINK1* mutations show a significant reduction in the level of endogenous parkin levels and are unable to initiate mitophagy due to dysfunction in ubiquitination pathways [141]. Furthermore, the level of Phosphorylated-Ser65-Ub signals are significantly reduced within iPSC-derived TH^+^ neurons with *PINK1* p.G411S mutation [125,142]. Consistent with this, the recruitment of parkin to mitochondria is impaired upon depolarization of mitochondria in *PINK1* mutated iPSC-derived DA neurons [137]. Overexpression of wild type *PINK1* in these DA neurons restored the translocation of parkin to mitochondria [137]. These studies highlighted the vital role of *PINK1* in mitochondrial function and pathogenesis of PD.

One of the major indicators of mitochondrial dysfunction is the generation of reactive oxygen species (ROS) which in turn leads to cell damage due to oxidative stress [143]. Significant damage to lipids, proteins, and nucleic acids have been identified in fibroblast of s carrying PINK mutation [144]. Further study demonstrated that *PINK1* deficiency results in an increased basal ROS in both the mitochondria (Figure 3) and cytoplasm, leading to increased oxidative stress in iPSC-derived DA neurons [145]. One of the key mechanisms in detoxification and prevention of ROS associated mitochondrial damage in the cytoplasm is the oxidation of glutathione (GSH). iPSC-derived neurons with *PINK1* Q456X mutation display reduced GSH levels and showed increase vulnerability after exposure to low concentrations of valinomycin, concanamycin A, MPP+ and hydrogen peroxide (all promotors of oxidative stress) in comparison to control neurons [84].

**Table 4:** PINK1-mutated iPSC-derived neuronal phenotypes.

Reference	Number of Cohorts	Type of Mutation	Cell Type	Phenotype
[137]	3 PD lines vs. 1 control line	c.1366C>T, c.509T>G	iPSC-derived DA neurons	1. Mitochondrial dysfunction
[84]	5 PD lines vs. 2 control lines	Q456X, R1441C	iPSC-derived DA neurons	1. Increase in oxidative stress 2. Mitochondrial dysfunction
[141]	1 PD line vs. 1 control line	V170G	iPSC-derived DA neurons	1. Impairement in mitophagy
[140]	7 PD lines vs. 5 control lines	Exon 4 or 7 deletion	iPSC-derived DA neurons	1. Dysregulation of *LRKK2* levels 2. Mitochondrial dysfunction

### 3.5. iPSC Modelling of GBA Mutation and Associated Phenotypes

The *GBA* gene is located on chromosome 1 (1q21) and encodes for a lysosomal glucocerebrosidase enzyme (GCase), hydrolysing the glucosylceramide (GlcCer) into ceramide and glucose [146]. *GBA* mutation was first associated with PD approximately 14 years ago as a result of a PD like phenotype in PD with Gaucher disease [147]. The onset of PD with *GBA* mutations have been reported to be 30% at 80 years, with 9.1% of *GBA* carriers develop PD [148]. The main *GBA* mutations are p.N370S and p.L444P, enhancing the Lewy bodies formation, leading to PD and dementia [149]. Both mutations exhibit a reduced GCase activity that trigger an abnormal accumulation of α-synuclein [146]. Moreover, *GBA* has a key role in mitochondrial function and autophagy [150].

#### 3.5.1. GBA Mutations Result in Disrupted Mitochondrial Function in iPSC-Derived Neurons

Mutations in all pN370S, pL444P, and RecNcil *GBA* iPSC-derived neurons have altered mitochondria morphology and function (Table 5). Morphological assessment using transmission electronic microscope (TEM) has demonstrated that mitochondrial have larger diameters and altered cristae in iPSCs-derived DA neurons in comparison to controls. Alterations have been additionally observed as a result of reduced oxygen consume rate (OCR) and complex I activity (CI) in *GBA* mutated neurons, associating with increased mtROS levels [151]. The ratio between long and short isoforms of fusion protein OPA1 are elevated in *GBA*-mutated iPSC-derived neurons, suggesting disruptions in mitophagy process, and subsequently in mitochondrial dynamics [151]. Furthermore, expression of mitophagy adaptor proteins such as BNIP3L/NIX have been significantly reduced in *GBA* mutated iPSC-derived neurons [151].

**Table 5:** GBA-mutated iPSC-derived neuronal phenotype.

Reference	Number of Cohorts	Type of Mutation	Cell Type	Phenotype
[150]	3 PD lines vs. 3 control lines	Heterozygous N370S	iPSC-derived DA neurons	1. Lysosomal dysfunction 2. Autophagy dysfunction 3. Endoplasmic Reticulum stress
[152]	1 PD line vs. 1 control line	Heterozygous N370S	iPSC-derived DA neurons	1. α- synuclein aggregation 2. Lipid dyshomeostasis
[151]	4 PD lines vs. 2 isogenic control lines	Heterozygous N370S, L444P and RecNciI	iPSC-derived DA neurons	1. Mitochondrial dysfunction 3. Lipid dyshomeostasis 4. Alteration in mitochondria and lysosome Colocalization 5. Endoplasmic reticulum stress
[153]		Homozygous N370S	iPSC-derived DA neurons	1.Dopamine dysregulation
[154]	1 PD line vs. 3 controls lines	Heterozygous N3070S	iPSC-derived DA neurons	1. Dopamine dysregulation 2. Elevated α-synuclein levels.
[155]	7 PD lines vs. 3 control lines	N370S	iPSC-derived DA neurons	1. Autophagic and autophagosome dysfunction. 2. Elevated α-synuclein levels
[156]	2 PD lines vs. 2 isogenic control lines	null GBA (CRISPR-Cas)	iPSC-derived cortical neurons	1. Reduction in Gcase activity 2. Lysosomal dysfunction
[157]	3 PD lines vs. 3 control lines	N370S	iPSC-derived DA neurons	1. Lysosomal dysfunction 2. Elevated α-synuclein levels

#### 3.5.2. GBA Mutations Result in ES Stress in iPSC-Derived Neurons

iPSC derived DA neurons with GBA mutations are vulnerable to increased endoplasmic reticulum (ER) stress, autophagic/lysosomal dysfunction, and eventually enlargement of lysosomal compartments [150]. This results from the accumulation of misfolded *GBA* in the ER, leading to the activation of the UPR, supported by an upregulation of Bip/GRP78, calreticulin and additional UPR-mediators such as PDI, calnexin and IRE1-alpha [150]. These findings suggest that accumulation of misfolded proteins can result in autophagy disturbances. In line with this, GBA-pN370S iPSCs-derived DA neurons display elevated level of LC3B-II, the lipidated form of the autophagosome marker, reflecting an increase in level of autophagosomes [150]. This was further confirmed in a study by Yang et al., in which the levels of LC3 and p62 were decreased, suggesting an impairment in autophagosome formation [155].

Impairments in lysosomal number and degradation processes are also associated with the reduced GCase activity in *GBA*-mutated cortical and DA neurons [150,156]. The level of cathepsin D, a lysosomal protease that interacts with GCase products is decreased, promoting a dysregulation in α-synuclein levels, disturbing lysosomal function in *GBA*-pN370S mutated iPSC-derived DA neurons [157]. The observed lysosome enlargement was found to be associated with an impairment of cargo degradation accompanied by elevated level of lysosomal markers LAMP1 and LAMP2 in iPSC-derived DA neurons with *GBA*-pN370S mutation [150]. Consistent with these findings, the electron microscopy images showed an accumulation of dense debris in lysosomes, suggesting abnormal lysosome clearance [150].

#### 3.5.3. GBA Mutations Result in α-Synuclein Aggregation in iPSC-Derived Neurons

The observed defects in autophagic/lysosome pathways enhance α-synuclein aggregation, impairing its release, as seen in GBA iPSCs-derived DA neurons [158]. Reduction of GCase activity by the accumulation of GlcCer enhance the formation of soluble toxic α-synuclein [152]. mRNA levels of α-synuclein in iPSCs-derived DA neurons with *GBA* mutation showed no significant difference compared to control, suggesting that *GBA* mutation only disrupt α-synuclein processing and not its transcription [154]. Accumulation of α-synuclein observed, impaired the degradation dopamine in *GBA*-pN370S heterozygous null neurons [156].

Degradation of DA neurons are controlled by monoamine oxidases (MAO). iPSC-derived DA neurons affected by *GBA*-pN370S have demonstrated an elevated activity of MAO [154]. These results are supported by an increase in MAO mRNA levels and protein, suggesting MAO upregulation in PD-affected patients [154]. Additionally, proteins implicated in dopamine level such as dopamine transporter DAT and VMAT2, were decreased in mRNA expression in iPSCs- derived DA neurons of GD with parkinsonism, against iPSCs-derived DA neurons of GD without parkinsonism [153]. Dopamine absorption studies in iPSCs-derived DA neurons shows that the level of dopamine in DA neurons with Gaucher Disease with parkinsonism is reduced [153].

### 3.6. iPSC Modelling of DJ-1 Mutation and Associated Phenotypes

Dj-1 is a small protein with 189 amino acid residues, usually forming homodimers having a key role in anti-oxidant activities as well as directly inhibiting α-synuclein aggregation [159]. Mutations in *DJ-1* have shown to cause early onset, autosomal recessive PD, either due to a base-pair deletion or a homozygous point mutation (L166P) [160,161]. The effects of mutations in *DJ-1* on the development of PD has not been extensively studied in iPSC-derived DA neurons (Table 6). Increased dopamine oxidation and oxidative stress have been observed in iPSC-derived DA neurons, triggering mitochondrial oxidative stress, leading to the inactivation of glucocerebrosidaes [160]. This inactivation in turn inhibits lysosomal functions, elevating the level of α-synuclein, a known phenotype observed in iPSC- derived neurons from PD [160]. A more recent study utilized hiPSC-derived DA neurons carrying *DJ-1* mutation and demonstrated a dysregulation in lysosomal proteins and activity [81,162,163].

## 4. Evidence That iPSC Models Confirm the Key Phenotypes Found in Human Pathology

DA degeneration is a key element in the development of PD, and increasingly the evidence for the direct or indirect involvement of mitochondrial dysfunction is growing. Numerous studies show that samples from PD patients show dysregulation in mitochondrial protein expression, glucose metabolism, and reduction in complex 1 activity [164,165,166]. The data to date shows that the DA neurons from the substantia nigra have a high energy demand to support their long, arborized axons and transmitter release sites, making these cells vulnerable to degeneration. This is supported by the evidence from iPSC-derived neurons with mutations in *LRRK2*, *PARK2,* and *SNCA*, where these differentiated neurons have shorter outgrowths and reduced numbers of neurites [58,167,168].

**Figure 4:**
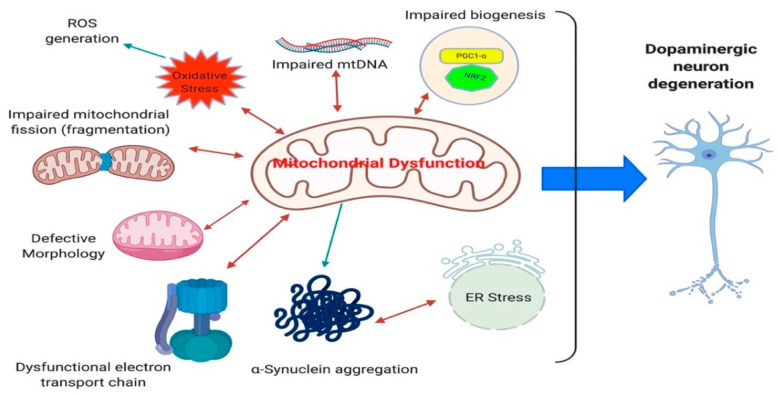
Pathways of mitochondrial dysfunction, a major cellular and clinical phenotype in PD. Mitochondrial dysfunction can result from impairment in mitochondrial fission, change in mitochondrial morphology, electron transport chain, increase in mtDNA, elevation in oxidative stress leading to reactive oxygen species generation, alteration in mitochondrial biogenesis and electron transport dysfunction. These can lead and associated with protein aggregation and eventual endoplasmic (ER) stress that ultimately results in degeneration of dopaminergic neurons that underlines the PD pathogenesis.

Alterations in both the functional and morphological aspects of mitochondria in iPSC-derived neurons have been demonstrated (Table 1, Table 2, Table 3, Table 4, Table 5 and Table 6). *PINK1*, *PARK2*, *LRRK2* G2019S, and *SNCA* mutations recapitulated the disease state very well, neurons present with an increase in fragmented mitochondria and an increase in mitochondrial content [48,57,82,111,140] (Figure 4). This suggests an overall reduction in the number of active functional mitochondria within iPSC-derived DA neurons and supports the energy drain as a causative agent in DA degeneration observed in patients. Furthermore, the morphology of mitochondria are shown to be swollen and disorganized in iPSC-derived DA neurons [48,151] (Figure 4). These alterations in mitochondrial content are in line with the idea that the neurons capacity to produce energy is compromised. As evidenced by the reduction in nicotinamide adenine dinucleotide (NAD^+^), in *LRRK2* G2019S iPSC-derived DA neurons [85]. Additionally, *GBA* mutations result in a reduction the NAD^+^/NADH ratio and treatment with NAD^+^ precursor nicotinamide riboside rescued the respiratory capacity in these neurons [151]. In the same domain, basal and maximal respiration capacity, ATP-linked respiration (Figure 4) and ATP production is significantly decreased in *SNCA*, *GBA*, *PINK1* and *LRRK2* G2019S iPSC-derived neurons [48,85,151,169]. All these data are consistent with the reduced levels of ATP synthase observed in substantia nigra of PD patients and re-enforces the quality of iPSC model systems to model the disease state as regards mitochondrial function [165].

Mitochondrial DNA (mtDNA) damage and accumulation of mtDNA deletions have been observed in substantia nigra of PD patients, which correlate with the cellular respiratory defects [170,171]. Moreover, a reduction in mtDNA copy number has been detected in cerebrospinal fluid of PD patients and in other patients with different neurodegenerative disorders [172,173]. Damage to mtDNA can lead to the breakdown of double-stranded DNA, causing the deletion and loss of several kilobases of mtDNA. This connection between double-strand breaks and mtDNA deletions has been studied in mouse models, developing PD-related behavioral phenotypes and degeneration of nigrostrial brain regions [174]. Increased mtDNA has been confirmed in neurons with *PINK1* and *LRRK2* G2019S mutations [82,137]. The mtDNA damage can explain the reported complex I deficiency observed in PD patients since the genes found in mtDNA encode components of the electron transport chain [175,176] (Figure 4). Studies in iPSC-derived neurons from different genetic background all confirm their association with the pathogenesis of PD. While these results suggest that mitochondrial alterations increase neuronal vulnerability and neuronal loss as seen in PD, it is still unclear whether these defects are causative agents or more downstream event in PD pathology.

An additional effect of accumulating somatic mtDNA deletions and reduction in ATP synthase is an increase in oxidative stress within DA neurons. Oxidative stress is one of the main factors involved in pathogenesis of PD and has been identified in post-mortem studies [177,178,179,180]. Increased reactive oxygen species generation (Figure 4) is evident due to an increase in respiration and metabolic demand, leading to an elevation in electron flux. In addition, reduced level of antioxidants has been shown in the brains of PD patients [181,182]. While these phenotypes showing increased oxidative stress could relate to mitochondrial dysfunction, an additional source could be the processing of dopamine by oxidases [183].

Oxidative stress is also one of the major phenotypes observed in many of the iPSC-derived DA neurons from different genetic backgrounds (Table 1, Table 2, Table 3, Table 4, Table 5 and Table 6). Increases in the level of carbonylated proteins are associated with both *LRRK2* and parkin mutations [118,184]. Additionally, the levels of proteins involved in dopamine oxidation (MAO-A and MAO-B) are significantly elevated in parkin, *SNCA* triplication, *GBA,* and *LRRK2* G2019S iPSC-derived DA neurons [56,79,118,154]. This phenotype can be rescued with the expression of MAO-A and -B, while the opposite occurs when parkin is overexpressed, further supporting the relationship between oxidative stress and PD pathology and the utility of iPSC models [118]. Consistent with this, the level of anti-oxidative genes Nrf2, NQO1 are upregulated in parkin mutated iPSC-derived neurons [111]. Furthermore, increased levels of ER stress and activation of unfolded protein response (UPR) have been demonstrated in substantia nigra of PD patients. ER stress is one of the least explored phenotypes which has been confirmed in *GBA*, *LRRK2* G2019S, and *SNCA* A53T iPSC-derived DA and cortical neurons [57,150,151,185]. The relationship between protein aggregation and cellular stress has been widely studied in post-mortem PD brains [177]. The utility of iPSC neurons makes them an ideal platform to better understand ER stress and study protein aggregation i.e., α-synuclein and eventual DA neuronal loss, which is central to PD pathology (Figure 4).

Oxidative stress is associated with increased uptake and accumulation of α-synuclein [186] as evidenced by elevated dendritic mitochondrial stress in DA neurons [187,188,189]. α-synuclein is the main protein expressed both in Lewy bodies and Lewy neurites; hence, it is important to understand the potential toxic effects of these aggregations in DA neurons. α-synuclein levels are increased in *LRRK2* G2019S, parkin, *PINK1*, *GBA*, *SNCA* triplication, and *DJ-1* iPSC-derived DA and cortical neurons (Table 1, Table 2, Table 3, Table 4, Table 5 and Table 6). Abnormal expression of α-synuclein [190] and its phosphorylation at serin 129 (pS129) are the most abundant form which is found in Lewy bodies [51,62,64,112,123]. Abnormal accumulation of the phosphorylated form of α-synuclein has also been observed in *LRRK2* G2019S, *SNCA* triplication, and parkin mutated iPSC-derived DA neurons [51,62,64,112,123]. While these data suggest a strong association of α-synuclein alterations in iPSC-derived DA neurons, it is still unclear if α-synuclein directly induces DA neuron toxicity.

### The Potential and Limitations of iPSC-Derived Neurons

The development of iPSC model systems provides immense potential in the realm of patient-specific disease modelling. This is particularly true in the case of Parkinson‘s disease, through the generation of DA neurons from PD patients. These cells facilitate the study of gene specific alterations, enabling researchers to characterize different functional and morphological deficiencies in patients own cells and should in the future allow more personalized, targeted therapies. The iPSC-derived neurons carrying gene specific mutations have been shown to display characteristics commonly found in human PD pathology which supports the further exploration of iPSC models. The next phase of this approach to disease modelling and therapy development is to fully characterize these cells and maximize the opportunity.

Many of the pathways highlighted here are directly associated with DA neuron vulnerability and intersect at the controlled generation and release of dopamine. iPSC-derived neurons are ideal for mechanistic-based drug development trials targeting the suppression of oxidative stress, maintenance of the DA morphology and reduction in aggregation/accumulation of α-synuclein. iPSC-derived neurons provide a platform for screening many drugs in order to ameliorate the detrimental phenotypes observed in PD patient derived neurons. For example, high throughput screening of a library of compounds revealed the ability of Isoxazole to specifically target MEFC2-PCG1**α** pathway, preventing neuronal damage [49]. Similarly, potential targets which are identified through screening can be validated using iPSC-derived neurons. In a study by Soldner et al. they validated a *SNCA* enhancer variant that is associated with PD with use of CRISPR/Cas9 edited iPSC-derived neurons [191]. iPSCs can be a potential model to bridge the gap between the pre-clinical and clinical trials studies, increasing the translation prospects of potential drug candidates, providing valuable opportunities which can eventually be extrapolated for therapeutic interventions for PD.

However, despite the many advantages, there are limitations in the use of iPSC neurons for modeling PD pathology in a dish. PD is associated with aging and it is a limitation in iPSC technology to generate an age matched model to more fully recapitulate the PD phenotypes. DA degeneration is a hallmark of PD which has not been observed in iPSC- derived DA neurons. A study by Miller et al., reported that the addition of progerin to parkin and *PINK1* iPSC-derived DA neurons can enhance apoptosis and shortening of the neurites, accelerating an aging process [192]. Progerin treatments in animal models have additionally shown reduction in survival of DA neurons [192]. Overexpression of progerin can improve the modelling of the late-onset PD. Telomerase inhibitor 2-[(E)-3-naphthalen-2-yl-but-2-enoylamino]-benzoic acid (BIBR1532) also exhibited a reduction in neurite branching increase in mitochondrial stress and DNA break in parkin iPSC-derived DA neurons [193]. While addition of these factors are beneficial in representing true DA neurons in iPSC modelling for PD phenotypes, it is, as yet difficult to distinguish the phenotypes observed from the factors-derived or normal DA neurons.

Another pitfall in iPSC modelling is the efficiency of different differentiation protocols and two-dimensional monolayer systems, which may not truly represent the complex neuronal signalling in vivo or in 3D in vitro scenarios. Protocols have been established for the rapid generation of organoids containing DA neurons, astrocytes and oligodendrocytes from iPSC-derived from PD patients [194,195]. Jo et al., have demonstrated electrically active mature DA neurons with the capability of dopamine production in 3D organoids [194]. Diminishing the genetic backgrounds has also been achieved with the use of CRISPR genome editing technology and use of isogenic controls. This not only eliminates the effect of genetic background but also minimizes the heterogeneity within iPSC lines. This can potentially provide new insights into understanding the complexity of DA degeneration process in PD. Furthermore, this may also help us to investigate and recapitulate other aspects of PD pathology such as neuroinflammation or Lewy bodies formation which as yet have not been established in iPSC-derived neurons. Very few studies using iPSC modelling have been used to study the contribution of astrocytes or microglia, which is a clear feature seen in PD.

## 5. Conclusions

Human PD-derived iPSCs are a powerful tool for improving our understanding of PD pathology and the underlying mechanism for DA degeneration and loss. There are still challenges in using iPSCs for the accurate recapitulation of PD phenotypes in humans. New 3D differentiation protocols, CRISPR genome editing technologies can advance the field for improving these challenges, paving the way towards a potential therapeutic target. iPSC technology can provide an exceptional opportunity not only to understand the PD pathology but additionally to understand the development of the disease, gaining invaluable information to facilitate the translation of research in a dish to clinical treatment.

## Figures and Tables

**Table 6:** *DJ-1*-mutated iPSC-derived neuronal phenotype.

Reference	Number of Cohorts	Type of Mutation	Cell Type	Phenotype
[162]	1 PD isogonic lines vs. control lines	*DJ-1*	iPSC-derived DA neurons	1. Increased oxidative stress 2. Mitochondrial dysfunction 3. Lysosomal dysfunction
[160]	3 PD lines vs. 2 control lines	c.192G>C	iPSC-derived DA neurons	1. Increased oxidative stress 2. lysosomal dysfunction 3. α -synuclein aggregation

## Data Availability

Not applicable.
